# Mental health disorders and recidivism among incarcerated adult offenders in a correctional facility in South Africa: A cluster analysis

**DOI:** 10.1371/journal.pone.0278194

**Published:** 2023-01-19

**Authors:** Kwanele Shishane, Johannes John-Langba, Eyitayo Onifade

**Affiliations:** 1 School of Society, Community and Health, University of Bedfordshire, Luton, England, United Kingdom; 2 School of Applied Human Sciences, University of KwaZulu-Natal, Durban, KwaZulu-Natal, South Africa; 3 Whitney M. Young Jr. School of Social Work, Clark Atlanta University, Atlanta, Georgia, United States of America; University of North Carolina at Chapel Hill, UNITED STATES

## Abstract

The contribution of mental illness, substance use, and appetitive aggression to recidivism has significant policy and practice implications. Offenders with untreated mental illness have a higher recidivism rate and a greater number of criminogenic risk factors than those without mental illness. Previous research has demonstrated that the likelihood of appetitive aggression increases in violent contexts where individuals perpetrate aggressive acts. Using the Ecological Systems Theory, this study investigated the association between mental health disorders and recidivism among incarcerated adult offenders in South Africa, and the intervening role of appetitive aggression and substance use. Using a cross-sectional quantitative research design, a sample of 280 incarcerated male and female adult offenders aged 18–35 with no known psychiatric disorders were sampled at a correctional facility in South Africa. The re-incarceration rate, mental health disorders, substance use, and appetitive aggression symptomology were assessed using the Hopkins symptoms checklist, the CRAFFT measure of substance use, and the appetitive aggression scale. Findings indicate a 32.4% recidivism rate (n = 82). Cluster analysis indicated that the combination of anxiety, depression, substance use, and appetitive aggression increased the likelihood of recidivism. Appetitive aggression median differences between clusters 2 and 3 played a key role in distinguishing recidivism risk among recidivist and non-recidivist participants. Chi-square analysis highlighted group differences in education levels among the established clusters [*x*^2^ (3, n = 217) = 12.832, p = .005, which is < .05] as well as group differences in the type of criminal offence [*x*^2^ (3, n = 187) = 24.362, p = .000, which is < .05] and cluster membership. Combined factors that increase the likelihood of recidivism provide a typology for classifying offenders based on particular recidivism risk determinants, which offers insights for developing tailored interventions that address a combination of factors.

## Introduction

Historically, if a community deemed particular behavior anomalous, unacceptable, or immoral, it was addressed through traditional courts as there were no prisons [1]. Traditional courts were managed by community chiefs and their assisters. For example, in the ‘Lekhotla’ [2,3], a community conflict resolution gathering generally practiced by the Sotho people, the chief would convene a hearing meeting including the offender, the victim, the offender, victim’s family, and community members. Traditional courts fostered peace, restoration, and reconciliation [2]. This approach to restorative justice was founded on the premise that when a community member violates another, the whole fabric of the community is affected [2].

Prisons, now referred to as correctional facilities in South Africa, did not originate from Africa; they were a Western system utilized to make people law-abiding citizens [1]. In those times, the convention of the law was unfair and unjust, and the courts’ judgments of offenders were often based on innate traits such as race [4]. This study recognizes the importance of person-first, non-stigmatizing language and therefore used the terms “offenders” and “correctional facilities” as required by the Department of Correctional Services in South Africa. In South Africa’s post-Apartheid era, social conditions promote violence and crime as normative while simultaneously using punitive measures to correct the behavior. Thus, the issue of incarceration and recidivism weakens structures that hold communities together and reduces employment opportunities, ultimately leading to poor social and economic development [5].

Recidivism indicates that there are challenges within the system where members of our society are trapped in the cycle of crime, and state strategies to address it are not as successful as intended. Consequently, recidivism remains a challenge [6]. The incarceration rate has expanded over the years, where correctional facilities are filled beyond capacity with startling overcrowding, leading to unfavorable living conditions for inmates [7]. Padayachee stated that recidivism ranges between 80%-94%, and many offenders recidivate in less than six months to a year [8]. Schoeman [9] posited that recidivism rates in South Africa range between 55%-95%. Similarly, Khwela [1] stated that general recidivism rates range between 50%-70% for offenders who recidivate within three years. The impact of educational programs on recidivism reduce the rate by at least 29% [1]. Karrim [10] estimated that 90% of South African offenders are repeat offenders. Studies investigating the nature and extent of recidivism and available statistics on recidivism in South Africa vary and are dispersed; however, estimates indicate that it is unacceptably high.

Studies have shown that recidivism is associated with several factors, such as substance use and mental illness [11,12], appetitive aggression [13], socioeconomic status, sexual orientation, age, population group/ethnicity, social support [14], low level of education, unemployment, antisocial peers [15], gang activity, and criminal history [16,17].

Bello [18] argued that recidivism has become the norm among African offenders and identified ten causes of recidivism in Africa: incorrigibility, failure of the sanction, lack of support in reintegration, failure of programs, peer pressure and other social provocations, economic stress, inability to attain employment, lack of education, mental health, and lack of support. Offenders living with mental illness also lack the capacity to appreciate their criminality. Therefore, they may be resistant to rehabilitative programs or any other measure taken in response to their crime [18]. As such, their propensity to re-offend is likely to continue until their mental health challenges are treated. If these mental health challenges remain untreated, offenders find themselves re-offending [12,18,19].

**Fig 1:**
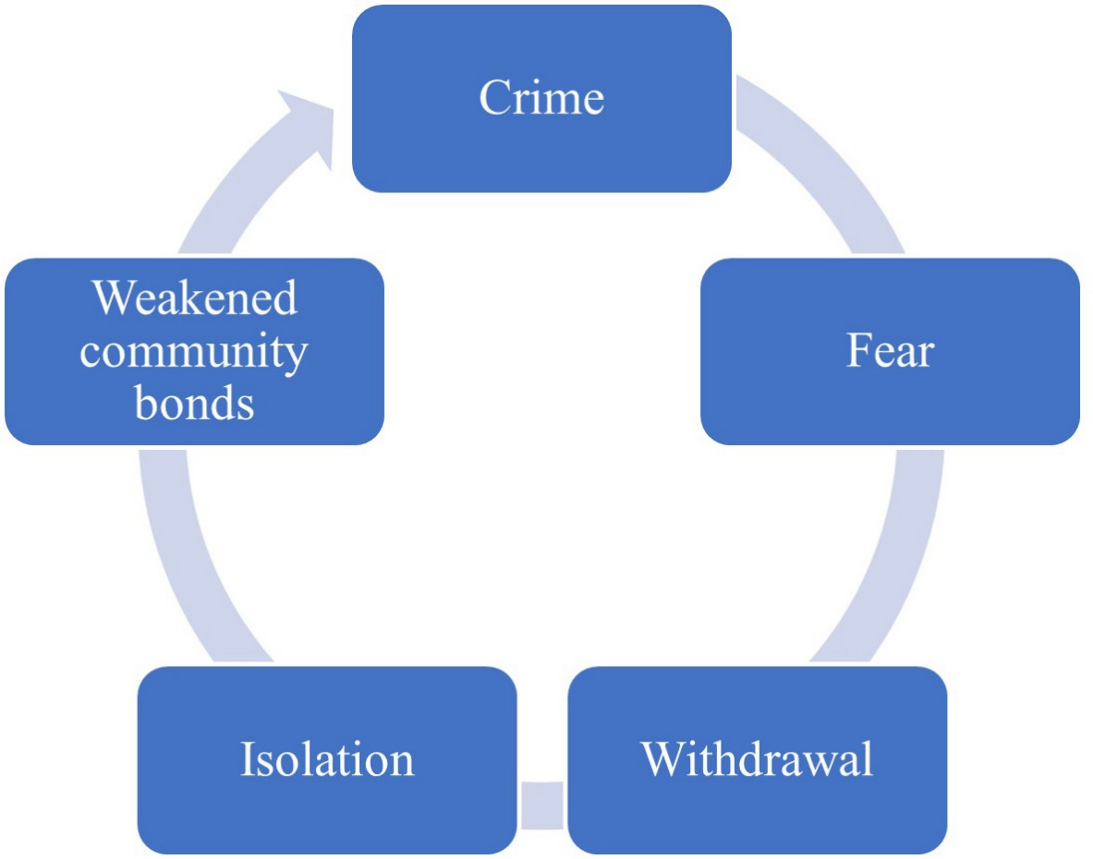
Crime cycle.

Naidoo and Mkize [20] assessed the prevalence of severe mental disorders in a South African correctional facility. The findings revealed a high prevalence of previously undetected psychiatric disorders among inmates. The lack of mental illness screening and treatment is concerning and suggests a need for examining the “criminalization of mental illness” hypothesis within South African correctional facilities. The lack of holistic approaches in investigating recidivism contributors among South African offenders creates and maintains fear [20]. As a result, communities at large become trapped in a downward spiral where crime increases fear, which increases isolation and lack of trust among community members, and, in turn, leads to more crime [21], as shown in Fig 1.

Research has demonstrated that the likelihood of appetitive aggression increases in violent contexts where individuals perpetrate aggressive acts. Appetitive aggression is based on the premise that the perpetration of violence is rewarding [22,23]. While the perpetrator willingly wants to harm the victim, the victim wants to avoid this behavior [24]. Generally, the expression of aggression and the degree to which aggressive behavior is accepted or punished are determined by a socializing environment [25]. Consequently, scholars argue that environmental processes are responsible for shaping aggressive behavior [13].

Low-income urban communities in South Africa are challenged by a cycle of violence in which young males predominantly play both victim and perpetrator roles [26]. Existing studies indicate that participants with higher Post-Traumatic Stress Disorder (PTSD) symptoms had lower psychosocial functioning and more concern about future threats. In contrast, participants with high appetitive aggression showed better functioning and fewer concerns about future threats [13]. Additionally, witnessed and self-experienced traumatic events predicted appetitive aggression, and higher appetitive aggression resulted in higher levels of violence perpetration and PTSD [26]. Furthermore, Sommer and colleages [27] found a positive relationship between exposure to traumatic events and PTSD symptom severity, appetitive aggression, the number of committed offenses, and substance use before perpetrating violence.

This study aimed to determine the association and group differences in mental health disorders (i.e., anxiety and depression), symptomology, and recidivism, and the intervening role of appetitive aggression and substance use in the association between mental health disorders and recidivism.

*The ecological systems theory as an explanation of social deviance and mental health in a post-apartheid context*. Theoretical models of crime underlying the deterrent approach to crime control often overlook the combined effect of mental illness, substance use, appetitive aggression, and the role of system responses, all of which are unique to a post-apartheid context. The Ecological System Theory addresses a person’s relationships with family, peers, and home from a micro-level [28,29]. For some imprisoned offenders, these relationships are strained, which eventually affects the offender’s reintegration upon release. In addition, from a mezzo level, the community reintegration of released offenders depends on community norms around criminal behavior, which influences whether or not the community stigmatizes, marginalizes, and excludes offenders because of their past criminal behavior or accepts them back into the community [29]. It also depends on post-prison rehabilitation services and support structures available to offenders within their communities [18].

Furthermore, from a macro perspective, an individual is affected by public policies, justice laws, government laws, economic systems, and social conditions [29]. For instance, society has a larger and more intense lens for black crime and thus is more likely to respond aggressively to black crime. In addition to social stigma, laws and systems make it difficult for offenders to obtain employment, limiting their ability to obtain basic needs [29]. Thus, this limitation directly impacts mental health, rehabilitation, and reintegration.

South Africa has a history of negative racialization, from the chronosystem perspective. For instance, many non-whites, particularly Blacks, were disadvantaged in their access to resources due to negative racialization [29]. Negative racialization and lack of access to resources have had rippling effects, leading to increased pressure to survive at the basic level of needs and the transfer of inter-generational trauma. These effects cause stress, leading to an increased risk of anxiety, mood disorders, and trauma, which are risk factors associated with deviance [29]. However, traditional explanations of black people’s criminal conduct exacerbate this problem by neglecting the contextual structures of an unequal society [29]. Therefore, in addition to the criminalization of mental illness, there is a case to be made for the continued criminalization of blackness in South Africa.

## Method

### Ethical considerations

Incarcerated offenders are considered vulnerable in research as they can be susceptible to coercion and undue influence. Therefore, it was pivotal to consider and apply ethics. The study received ethical approval from the University of Cape Town’s research ethics committee and the Department of Correctional Services research ethics board. The researcher randomly selected participants. Participation was voluntary, which was confirmed by obtaining signed informed consent forms from participants. For participants who could not read and write, verbal consent and an informed consent form marked with an X to indicate consent were obtained. Before completing the questionnaire, participants were briefed on the purpose of the research and their rights to participate, refuse to participate, and withdraw without any consequence. They were also asked not to write any identifying information such as names, ID numbers, or incarceration IDs on the form to ensure anonymity. In addition, participants were informed that their information would remain confidential. The study was not intrusive as it was self-administered and involved an interview with the principal investigator for participants who could not read and write.

### Study site

The research was conducted at the Durban-Westville correctional facility in KwaZulu-Natal, South Africa. The correctional facility is divided into five mediums, i.e., units where offenders are housed according to the security risk, which ranges from minimum to maximum. According to Singh [30], medium A houses awaiting trial detainees, medium B is a maximum-security medium for sentenced male offenders, medium C is for short-to-medium-term sentenced offenders, medium D is for juveniles, and medium E is for female offenders.

### Research design

A cross-sectional quantitative exploratory research design was used to investigate the association between mental health disorders and recidivism among incarcerated adult offenders in South Africa, as well as the intervening role of appetitive aggression and substance use.

#### Hypothesis

**Fig 2:**
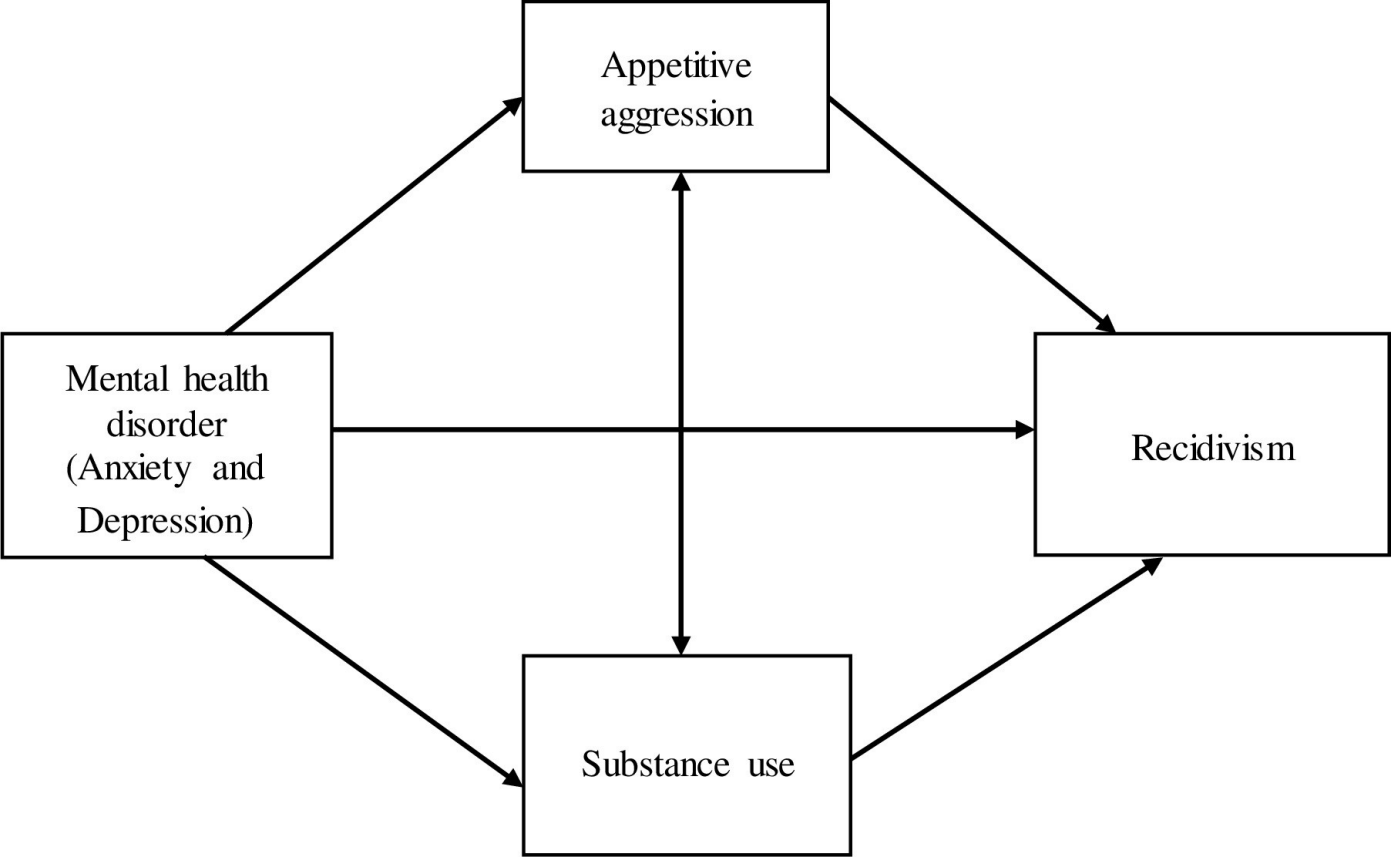
A hypothesized model of the central study variables.

We hypothesized that the interaction between appetitive aggression and substance use would have an effect on the relationship between mental health disorders and recidivism (See Fig 2).

### Instrumentation, variables, and measures

The research instrument was written in English and IsiZulu, as the majority of the population in KwaZulu-Natal speaks IsiZulu. Therefore, it was anticipated that most of the offender population spoke IsiZulu. The questionnaire was translated from English into IsiZulu by a competent bilingual IsiZulu speaker. The translated IsiZulu questionnaire was back-translated into English by a competent bilingual English speaker who had not seen the original English version of the questionnaire. The original and back-translated English version of the questionnaire was reviewed and corrected for errors and inconsistencies and thereafter finalized for pre-testing. The final questionnaire was piloted among offenders who would not form part of the study population. The questionnaire solicited participants’ socio-demographic information and history of incarceration. It included measures that measured the independent, intervening, and dependent variables.

#### Independent variables

Mental health disorders: this study focused on depression and anxiety as defined in the Diagnostic and Statistical Manual (DSM-IV-R) of the American Psychiatric Association [31]. Depression and anxiety symptomology were measured using the Hopkins Symptoms Checklist (HSCL). Parloff, Kelman, and Frank initially developed the measure at Johns Hopkins University in 1954 for use in primary care settings. The measure has also been used in various settings, including with offender populations [32]. The HSCL-25 consists of two parts: Part 1 has ten items for anxiety symptoms, e.g., “Suddenly scared for no reason”; Part 2 has 15 items for depression symptoms, e.g., “Feeling hopeless about the future.” Participants had to rate each item on a 4-point Likert scale (“Not at all,” “A little,” “Quite a bit,” and “Extremely” rated 1 to 4, respectively). In this study, the reliability analysis showed that the HSCL Cronbach’s alpha on standardized items was .920, and the Cronbach’s alpha for the anxiety and depression sub-scales were .861 and .881, respectively.

#### Intervening variables

Appetitive aggression: refers to a disposition towards a lust for aggressive behavior [33], measured using an adapted version of the appetitive aggression scale [34]. The original version of this scale measures attraction to violence, and in this study, it was coined as the attraction to crime and not just violent crimes. The scale consists of 15 statements on a 5-point Likert scale, ranging from totally disagree (0), disagree (1), neither agree nor disagree (2), agree (3), and totally agree (4). For example, “Is it exciting for you if you make a person really suffer?” The internal consistency of the modified version of the appetitive aggression scale was assessed using Cronbach’s alpha. The reliability assessment results show that it was highly reliable with this population (Cronbach’s alpha = .803).

#### Substance use

Substance use was measured using the Car, Relax, Alone, Forget, Family or Friends, Trouble (CRAFFT) measure of substance use. The CRAFFT measures alcohol and substance (drug) use. Knight et al., [35] developed the CRAFFT measure at the Center for Adolescent Substance Abuse Research (CeASAR) at Boston Children’s Hospital. It is a dichotomous measure with nine items and no (0) and yes (1) as response options. For example, “Does your FAMILY or FRIENDS ever tell you that you should cut down on your drinking or drug use?” For all items, the greater value indicates problematic alcohol or substance use. In this study, the reliability analysis of this measure showed a Cronbach’s alpha of .825.

#### Dependent variable

Recidivism: constructed from the Latin word recidiv or recidere, which means “to fall back” or “to relapse” [36], refers to re-incarceration and is measured by the number of times offenders have been sentenced to a correctional facility. Offenders who have been re-incarcerated and sentenced to a correctional facility more than once, i.e., two or more times, are considered to have recidivated.

### Sampling

This study used multi-stage cluster sampling, where participants were systematically selected from existing correctional facility mediums treated as clusters, namely, medium B, D, and E. This sampling procedure was used to get a reasonably distributed representation within clusters. Juveniles below the age of 18 were excluded from participating in the study. A list of 2775 offenders housed in mediums B, D, and E was obtained prior to data collection. Of the 2775 offenders list provided to the researcher, 1614 were eligible to be selected to participate based on the age criterion, i.e., 18–35 years. Every 4th person (Kth term) within the clusters was selected to participate, which meant 424 offenders could participate in the research. The target sample of 316 participants was obtained from the 424 selected. Three hundred and sixteen adult offenders aged 18 to 35 participated in the study.

### Data collection approach

A self-administered, hard-copy structured questionnaire was used to collect data. Data were collected within the correctional facility between June and August 2018. In compliance with correctional facility rules, some correctional facility wardens had to be present at the data collection center to ensure the safety of the offenders and the researcher. Before completing the questionnaire, the researcher explained the information on the participant information sheet and allowed for questions. The researcher also explained the purpose of the study and clarified that participation was entirely voluntary and would have no bearing on the offender’s sentence.

Instructions for completing the questionnaire were provided. In cases where participants could not read or write, one-to-one interviews were held with the researcher in the presence of a correctional facility warden who was within visual but not hearing distance. After completing the questionnaire, all pens and questionnaires were collected from the participants. A debriefing session was held, during which interested participants shared their questionnaire-completion experiences and had the opportunity to ask questions. Finally, if participants experienced or later experienced any psychological or emotional distress from participating in the study, the researcher signposted them to mental health support structures within the correctional facility. At the time of data collection, the researcher liaised with the correctional facility psychologist and three social work probation officers who would provide psychosocial support to participants during the study period.

### Data analysis

Data were analyzed using the Statistical Package for Social Science (SPSS v26). While 316 participants participated in the study, this analysis is based on 280 participants, as 36 participants with invalid or missing data were removed from the dataset. We began by conducting the normality test on the dependent variable, i.e., recidivism, and all central study variables. Normality was tested using the Shapiro-Wilk test. Findings revealed that the assumption of normality was violated, thus indicating that the data is not normally distributed and should be analyzed with non-parametric tests. The normality test was followed by a descriptive analysis to depict the participants’ socio-demographic data. Prior to descriptive analysis, central study variables were transformed into categorical variables. After that, a two-step cluster analysis was used to identify cluster membership. “Cluster analysis is a procedure by which individuals are sorted into meaningful and relatively homogenous groups given their patterns of scores across several dimensions” [37]. The cluster analysis was first conducted by running pre-clustering and then a hierarchical method. The anxiety and depression variables were entered as input variables, and appetitive aggression and substance use were entered as evaluation fields. The change in quartile scores and median (IQR) scores between clusters on a box plot determined cluster differences, where the median marks the mid-point that divides the box into groups. Notably, the naming of the clusters attempts to reflect the characteristics of each empirically determined group. Finally, using the p < .05 threshold, Chi-Square analysis was used to determine group differences between socio-demographic variables and the clusters.

## Results

### Demographic characteristics of participants

Table 1 presents participants’ demographic characteristics. Their minimum and maximum age were 19 and 35, respectively. The majority of participants (mode) were aged 32, with a mean of 30 and a standard deviation of 3.8. Most participants have had secondary and post-secondary education (n = 211, 81.2%). They were mostly Black/African (n = 245, 91.4%). In terms of gender, most participants were male (n = 251, 93.0%). Most participants are not married (n = 264, 97.8%). Participants reported having biological children (n = 177, 65.1%). As expected, most participants were from KwaZulu-Natal (n = 237, 87.1%). In terms of the history of incarceration, the age of the first arrest ranged from 15 (n = 4, 1.9%) to 35 years (n = 1, .5%), the mode was 25 (n = 22, 10.6%), the median was 24 (n = 20, 9.7%), the mean was 23.8, and the standard deviation was 4.4. The most predominant crimes committed were property and non-violent crimes (n = 113, 54.1%), followed by violent crimes (n = 96, 45.9%).

**Table 1:** Demographic characteristics of participants.

Socio-Demographics	Frequency	Percent
**Highest level of education (n = 260)**		
None or Primary School Education	49	18.8
Secondary or Post-secondary school	211	81.2
**Population group (n = 268)**		
Black	245	91.4
Coloured	17	6.3
White	2	.7
Indian	4	1.5
**Sex (n = 270)**		
Male	251	93.0
Female	19	7.0
**Marital status (n = 270)**		
No	264	97.8
Yes	6	2.2
**Province of origin (n = 272)**		
KwaZulu-Natal	237	87.1
Gauteng	14	5.1
Eastern Cape	14	5.1
Limpopo	1	.4
Northern Cape	2	.7
Not from South Africa	3	1.1
**Type of criminal offense (n = 209)**		
Violent Crimes	96	45.9
Property and Non-Violent Crimes	113	54.1
**Recidivism (n = 253)**		
Not-Recidivated	171	67.6
Recidivated	82	32.4

### The prevalence of recidivism

The majority of participants have been sentenced once (n = 171, 67.6%), twice (n = 57, 22.5%), three times (n = 17, 6.7%), four times (n = 4, 1.6%), five times (n = 3, 1.2%), and more than five times (n = 1, 4%). When the recidivism variable was transformed into a dichotomous variable to depict recidivist participants from non-recidivist participants, findings indicated that 171 participants (n = 171, 67.6%) were non-recidivists, while 82 (n = 82, 32.4%) had recidivated. Therefore, the rate of recidivism among this offender population is 32.4%.

### Cluster analysis of mental health disorders, appetitive aggression, substance use, and recidivism

Using SPSS v. 26, a two-step cluster analysis of the factors (i.e., anxiety, depression, substance use, and appetitive aggression) was used to identify distinct clusters. Of the 280 participants, the researcher identified a special population that did not report their substance use and appetitive aggression. Therefore, due to missing values, cluster analysis findings are presented for 230 (n = 230) valid cases. Of the 230 cases, four clusters were generated on SPSS.

**Fig 3:**
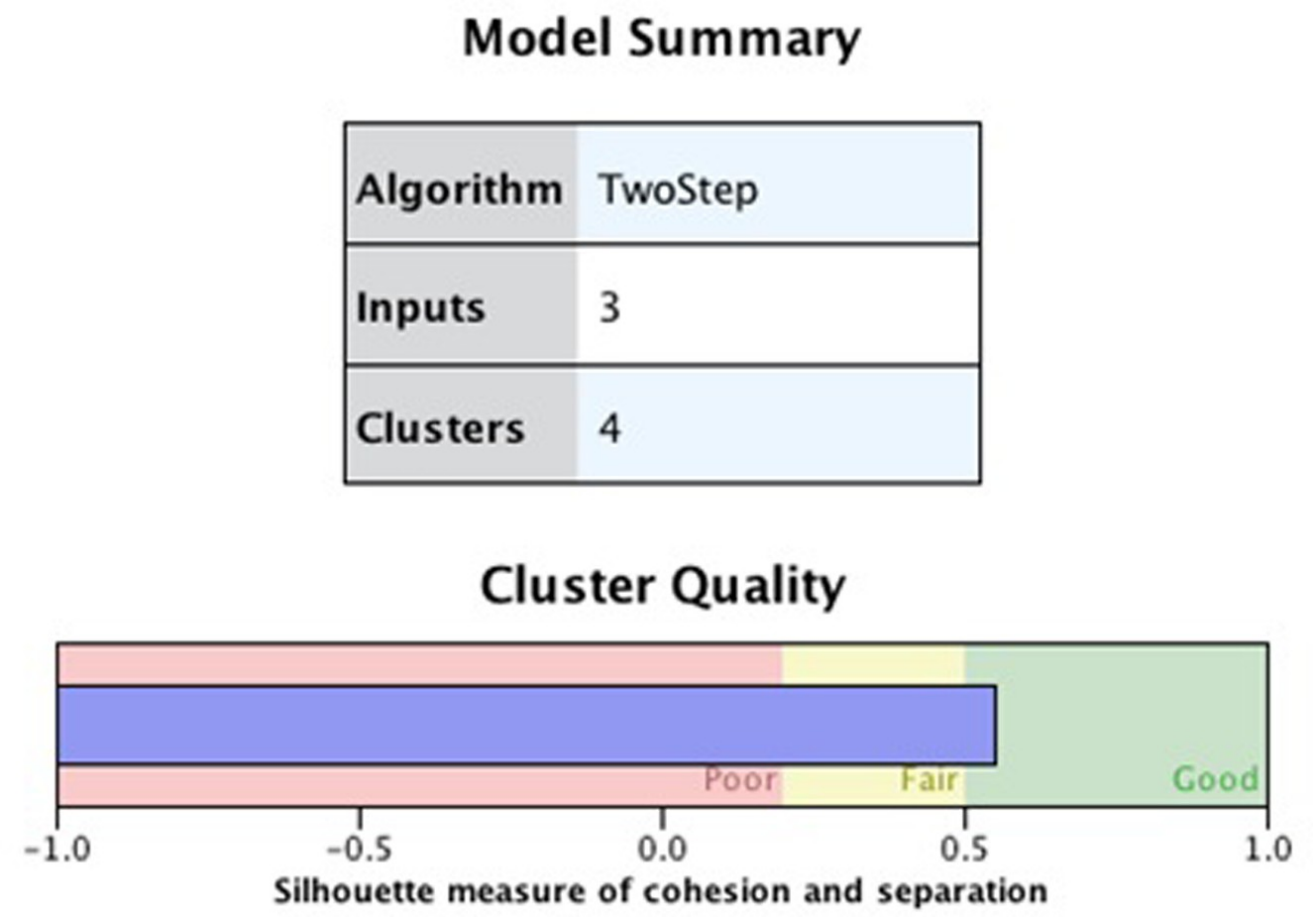
Silhouette measure of cohesion and separation diagram.

The naming of the clusters aims to reflect the characteristics of each cluster, depict the risk level, and allow for comparison. The Silhouette measure of cohesion and separation was 0.6, indicating that the cluster quality was significant and the model fits the data (see Fig 3).

#### Clusters overall whiskers box plot analysis

For all clusters, the anxiety box plot indicates an overall interquartile range (IQR) of 13.99 for Q1 (about 25% of the data) and 23.02 for Q3 (about 75% of the data), with an overall median of 17.96. Also, the overall IQR for depression was Q1 = 26.00 (representing about 25% of the data) and Q3 = 40.76 (representing about 75% of the data), with an overall median of 32.08. The overall IQR for appetitive aggression was Q1 = 6.05 and Q3 = 18.77, with an overall median of 12.04. Additionally, the overall IQR for substance use was Q1 = 1.01, while Q3 = 6.50, with an overall median of 4.00. Clusters were classified as low or high risk, depending on whether they fell above or below the overall median of each central study variable.

#### Cluster 1 “low risk”

**Fig 4:**
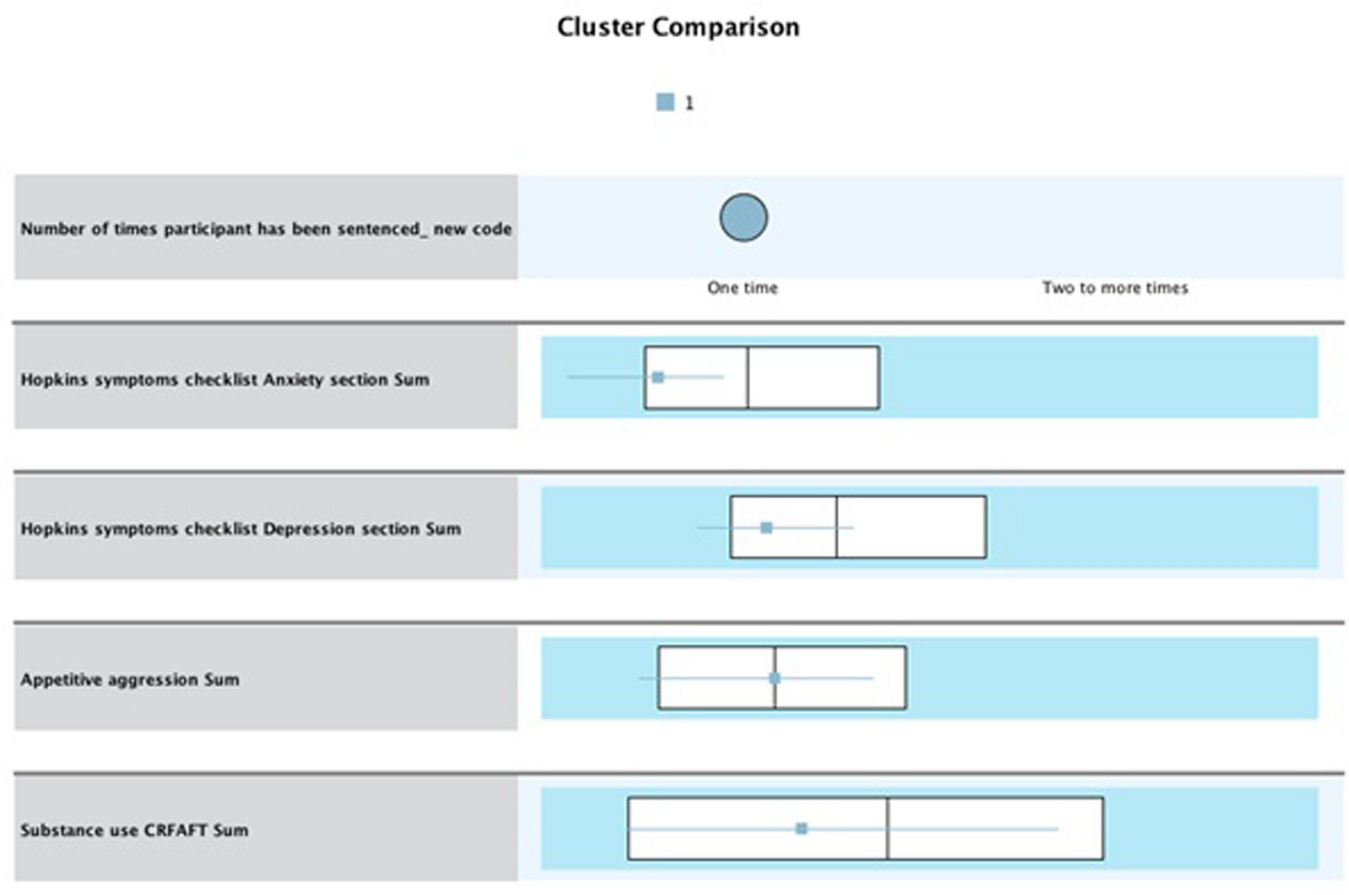
Cluster 1 “low risk” (non-recidivist, n = 98) whiskers box plot of values associated with each variable.

Cluster 1 constituted 98 participants (n = 98, 42.6%). These participants have not recidivated. In this cluster, the IQR for anxiety is Q1 = 11.04 (about 25% of the data), while Q3 = 17.01 (representing about 75% of the data), with a median of 14.51. Thus, participants in this cluster scored below the overall median for anxiety. For depression, the IQR is Q1 = 24.03 (representing about 25% of the data), while Q3 = 33.04 (representing about 75% of the data), with a median of 28.04. Thus, participants in this cluster scored below the overall median for depression. For appetitive aggression, the IQR is Q1 = 5.06 and Q3 = 17.04, with a median of 12.01. For substance use, participants scored below the overall median at 3.02 (see Fig 4).

#### Cluster 2 “high risk”

**Fig 5:**
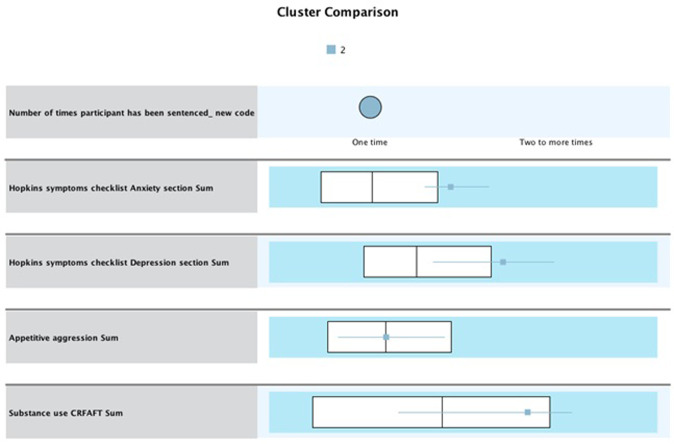
Cluster 2 “high risk” (non-recidivist, n = 61) whiskers box plot of values associated with each variable.

Cluster 2 constituted 61 of the offender population (n = 61, 26.5%). This cluster also consists of participants who have not recidivated. In this cluster, the IQR for anxiety is Q1 = 22.04 (representing about 25% of the data), while Q3 = 26.98 (representing about 75% of the data), with a median of 24.02. Thus, participants in this cluster scored higher than the overall median of 17.96 and higher than the Q3 = 23.02. For depression, the IQR is Q1 = 34.01, while Q3 = 47.96, with a median of 42.11. Thus, participants in this cluster scored higher than the overall median of 32.08 and higher than the Q3 range of 40.76. For appetitive aggression, findings indicate an IQR of Q1 = 7.10 and Q3 = 18.05, with a median of 12.04. For substance use, participants scored above the median at 5.99 (see Fig 5). This cluster presented with a combined high mental health and substance use problem, which may explain criminal behavior. The incidence of substance use in this cluster also suggests that offenders may be self-medicating with substances.

#### Cluster 3 “high risk”

**Fig 6:**
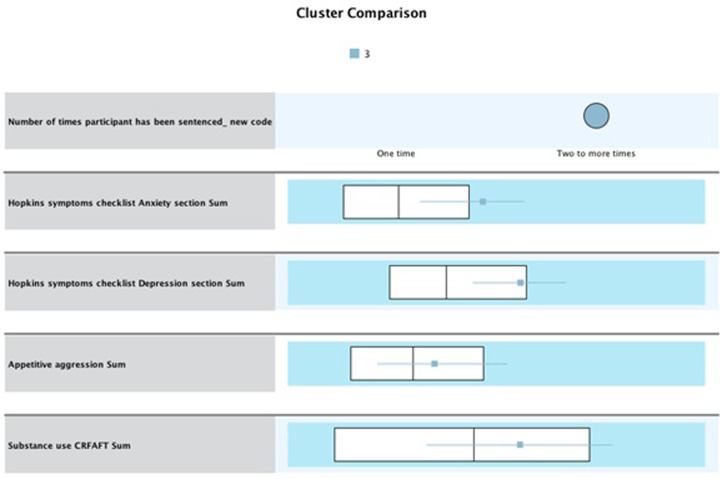
Cluster 3 “high risk” (recidivist, n = 39) whiskers box plot of values associated with each variable.

Cluster 3 consisted of 39 participants (n = 39, 17.0%). Participants within this cluster have recidivated. The IQR for anxiety is Q1 = 19.56, while Q3 = 26.96, with a median of 24.02. Thus, participants in this cluster scored higher than the overall median of 17.96 and higher than the Q3 range of 23.02. For depression, the IQR is Q1 = 34.98, while Q3 = 44.98, with a median of 40.09. Thus, participants in this cluster scored higher than the overall median of 32.08 and were within the higher Q3 range. For appetitive aggression, findings indicate an IQR of Q1 = 8.60 and Q3 = 21.02, with a median of 14.07. Although small, the difference is higher than the overall median of 12.04, and the median is higher in this cluster compared to cluster 2. Participants scored above the overall median for substance use at 5.01 and within the higher Q3 range of 6.50 (see Fig 6). Like cluster 2, this cluster had a high level of mental health and substance use problems. Although small, the median differences in appetitive aggression between clusters 2 and 3 show that the added effect of appetitive aggression may explain this cluster’s recidivism.

#### Cluster 4 “low risk”

**Fig 7:**
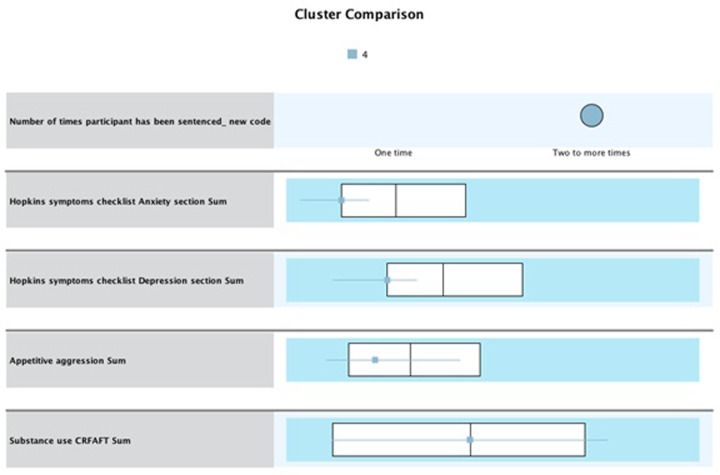
Cluster 4 “low risk” (recidivist, n = 32) whiskers box plot of values associated with each variable.

Cluster 4 consisted of 32 participants (n = 32, 9%). Participants in this cluster have recidivated. The IQR for anxiety is Q1 = 11.02, while Q3 = 16.00, with a median of 14.01. Thus, participants in this cluster scored lower than the overall median of 17.96 and were within the lower range quartile. For depression, the IQR for Q1 = 20.05, while Q3 = 29.26, with a median of 26.00. Thus, participants in this cluster scored lower than the overall median of 32.08 and within the lower Q1 range. For appetitive aggression, findings show an IQR of Q1 = 3.85 and Q3 = 16.79, with a median of 8.60. Thus, participants in this cluster scored lower than the overall median of 12.04 and within the lower Q1 range. For substance use, the participant’s IQR is Q1 = 1.01 while Q3 = 7.00, with a median of 4.00 (see Fig 7). While this cluster presents with mental stability, substance use may explain re-offending behavior.

Overall, the four clusters mirror each other (cluster 1 and cluster 4 vs. cluster 2 and cluster 3). Although participants in clusters 2 and 3 had similar risk levels, there were differences in offending behavior, where one cluster recidivated while the other did not. Although mental health is not an explanatory variable, appetitive aggression played a key role in distinguishing clusters 2 and 3. Although the difference is small, it exists nonetheless and requires further research on appetitive aggression as a risk factor for recidivism.

### Cross tabulation and Chi-Square test of independence between demographic variables and clusters

Cross tabs and the Chi-Square test of independence were calculated for all socio-demographic characteristics and the clusters to determine group differences. No statistically significant difference was found between the majority of the socio-demographic characteristics and the clusters, except with education and the type of criminal offense.

**Fig 8:**
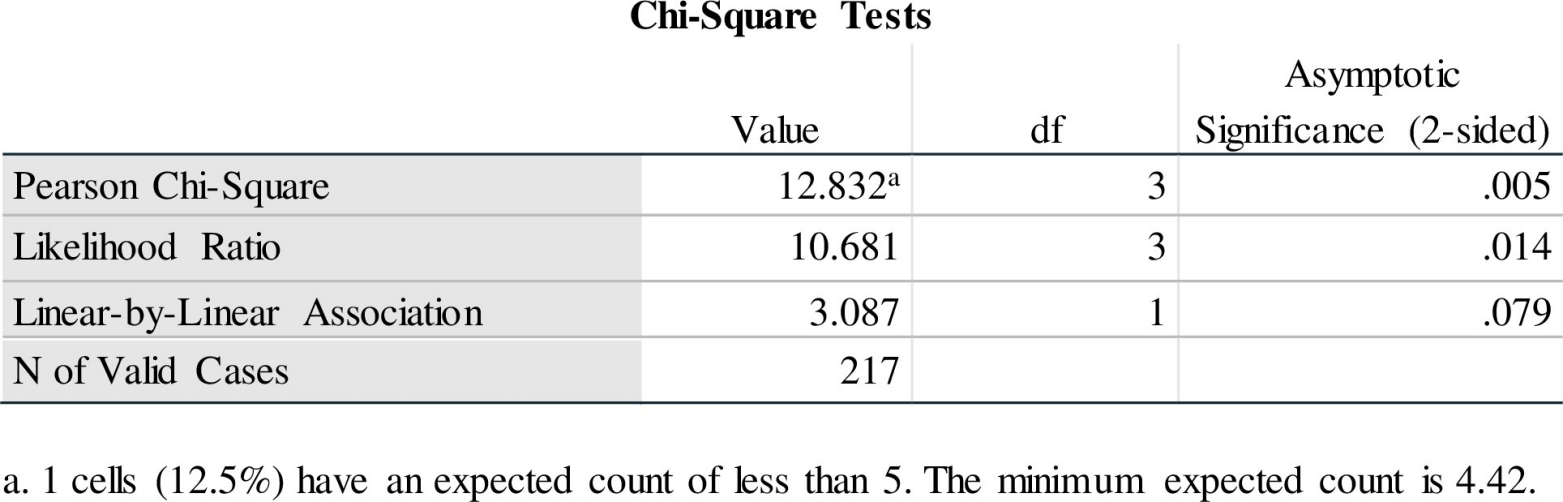
Chi-square test of cluster membership based on education level.

Crosstabs were conducted to determine differences in cluster membership based on participants’ level of formal education, which was recoded into a dichotomous variable (none or primary school education and secondary or post-secondary education). Findings indicated that cluster 1 had higher levels of formal education than other clusters. A Chi-Square test of independence was conducted to compare group differences in education levels among the four established clusters. A statistically significant difference was found [*x*^2^ (3, n = 217) = 12.832, p = .005, which is < .05]. Thus, cluster membership is not independent of the level of education (see Fig 8).

**Fig 9:**
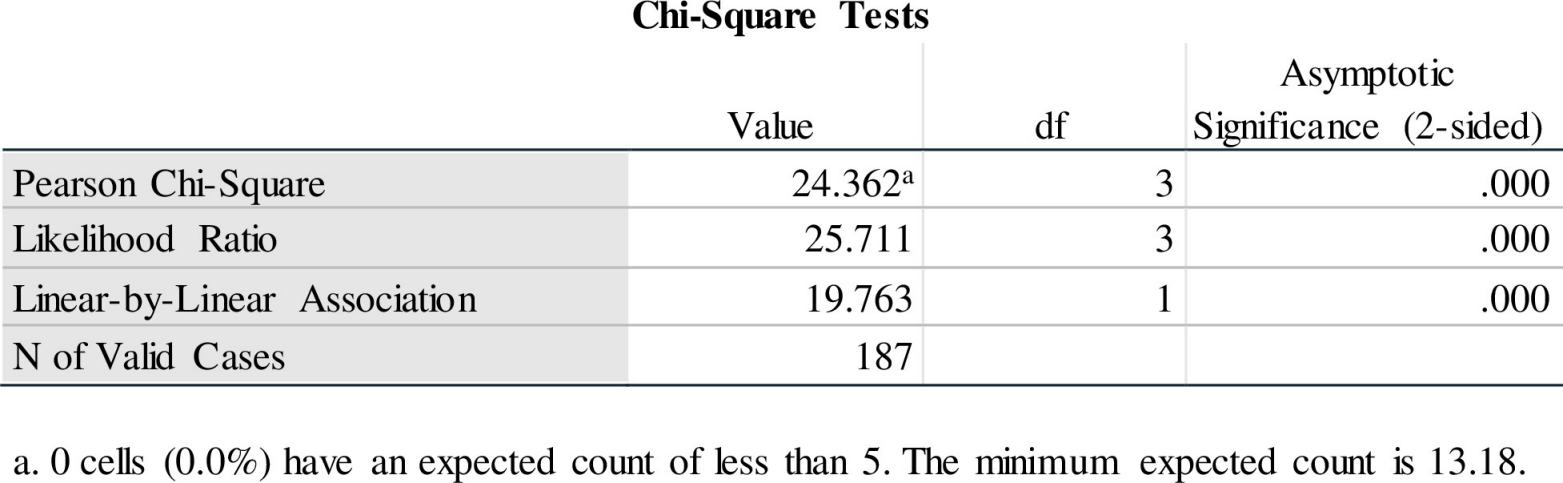
Chi-square test of cluster membership based on offense type.

Crosstabs were conducted to determine differences in cluster membership based on participants’ criminal offense types. Offense type was recoded into a dichotomous variable, i.e., violent crimes and property or non-violent crimes. Findings indicated that property or non-violent crimes were the most predominantly committed offenses, and the majority of participants who committed property or non-violent crimes were in cluster 1. A chi-square test of independence was conducted to determine group differences between crime type and cluster membership. A significant difference was found [*x*^2^ (3, n = 187) = 24.362, p = .000, which is < .05]. Thus, cluster membership is not independent of criminal offense type (see Fig 9).

## Discussion

This study aimed to quantitatively explore the association between mental health disorders and recidivism among incarcerated adult offenders in South Africa, as well as the intervening roles of substance use and appetitive aggression. It found a 32.4% rate of recidivism. While South African studies present varying recidivism rates, one that is relatively close to what this study found is a 29% recidivism rate [1]. A combination of anxiety symptomology, depression symptomology, substance use, and appetitive aggression influenced recidivism, where appetitive aggression played a key in distinguishing recidivism risk among recidivist and non-recidivist participants. Cluster 2 had not recidivated, whereas cluster 3 had recidivated and scored above the overall appetitive aggression IQR (median) score.

While the appetitive aggression scale was adapted to measure crime in general rather than violent crime, the predictive influence of appetitive aggression on re-offending behavior is consistent with previous research associating defiant behavior with mental health disorders and appetitive aggression [11,15,26,27]. It should also be noted that the cluster analysis findings may be impacted by 30 missing cases that were excluded from the analysis.

Furthermore, while offenders may share similar risk levels, they can have different offending patterns, as demonstrated in this study by clusters 2 and 3, which mirrored each other at similar risk levels. However, one cluster had recidivated while the other had not. The results highlight a need for further research on the role of appetitive aggression as a recidivism risk determinant with larger and more diverse incarcerated offender populations. Previous research has shown that in South Africa’s low-income urban communities challenged by a cycle of violence, young males are predominantly in the roles of both victim and perpetrator. Where exposure to violence, whether witnessed or experienced, is associated with higher PTSD symptoms, which are associated with lower psychosocial functioning and more concerns about the future, young males with appetitive aggression show better functioning and fewer concerns about future threats, given their appetite for aggressive behavior [13,26]. Thus, more research on appetitive aggression and defiant behavior would provide valuable information.

The presence of undetected mental health disorder symptomology among participants is also concerning because the first-ever study conducted in South Africa to determine the prevalence of mental disorders at the same correctional facility where this study was conducted revealed that offenders presented major depressive and psychotic disorders that were neither diagnosed nor treated [20].

Additional research findings attained through a chi-square test of independence indicated a significant difference in educational attainment and cluster membership. Most participants had secondary or post-secondary education compared to those without formal or primary school education. Such findings are consistent with previous research [38] that identified a low level of education as a significant predictor of offending behavior and recidivism in South Africa. These findings are also consistent with previous research indicating that offenders often lack education, which results in challenges to securing employment, which ultimately leads to a lack of economic independence [39,40]. Past research also asserts that offenders released back into the community after incarceration often recidivate due to their inability to secure employment as a result of their low levels of education [11].

Chi-square analysis also showed a significant difference in the type of criminal offence a participant was sentenced for and cluster membership, where property or non-violent crimes were the most committed crimes compared to other crimes. Such findings have implications for social work practice and social justice rehabilitation programs as they are consistent with previous research [41–44].

## Conclusion

The Department of Correctional Services faces myriad challenges in the rehabilitation and reintegration of offenders, which requires additional research to address these challenges. The most important aspect of this study is the comparison of anxiety, depression, appetitive aggression, and substance use between the non-recidivist and recidivist groups. Despite a small but significant difference in significance, appetitive aggression played a key role in distinguishing recidivism among the non-recidivist and recidivist groups. Future studies should explore the likelihood of re-incarceration based on a combination of risk factors. Additionally, studies exploring the effects of appetitive aggression on recidivism with larger and more diverse adult offender population sizes and at multiple sites must be conducted to further validate the instrument within this context.

The findings of this study show a high rate of mental health problems among this offender population. As a result, inferences can be made about the criminalization of mental illness, which cannot be confirmed by the current data but has been confirmed by previous research [20], indicating the failure to diagnose and treat offenders within correctional facilities. Thus, future studies can explore the criminalization of the mental illness hypothesis as a proxy for the criminalization of the blackness hypothesis.

This study examined offenders’ mental health, appetitive aggression, and substance use. However, it did not explore the criminal justice system as adjudicators of offenders who may or may not have sensitivity toward mental health such that the ecology of offenders who encounter the system is taken into account. The focus is mainly on system responses, which tend to assume that all criminal behaviour requires a formal response from the justice system and focus on the seriousness of a presented crime to direct decision-making rather than the offender’s risk needs [45,46].

Risk-Need Responsivity helps identify and reduce criminogenic risk needs [45]. While the model has been critiqued and acknowledged revisions made, scholars contend it is a sound and empirically tested model for responding to recidivism [47]. This approach could potentially reduce the overuse of valuable police, court, and correctional facility resources and services on individuals who could benefit from other services, such as diversion programs for low-risk offenders [48]. In this regard, recommendations are made for testing the effectiveness of the Risk-Need Responsivity (RNR) Model in the South African justice system.

Overall, this research concludes that intervention strategies that address recidivism risk factors need to integrate a multidimensional understanding of the role of mental health on recidivism and appetitive aggression, education, and offense type. Addressing adult offenders’ mental health needs and education may significantly improve their mental health status, making it possible for them to appreciate the crime committed and effectively respond to rehabilitation. Education programs offered within correctional facilities will remain ineffective in the absence of structured programs that launch released offenders into employment, as having a criminal record often limits their employment opportunities. Thus, a structured program would help influence behavior change and reduce recidivism through effective restoration and reintegration.

## Supporting information

S1 File (PDF)
