# Supplementary material for: Mental health disorders and recidivism among incarcerated adult offenders in a correctional facility in South Africa: A cluster analysis
Source: PLoS One. 2023 Jan 19;18(1):e0278194. doi: 10.1371/journal.pone.0278194 (PMC9851546; doi:10.1371/journal.pone.0278194)
Supplement: S1 File — (PDF) [file pone.0278194.s001.pdf]

Date: October 13, 2022

CERTIFICATE OF ENGLISH EDITING

This is to certify that the manuscript:

Mental health disorders and recidivism among incarcerated adult offenders in a correctional facility in South Africa: A cluster analysis

By the authors:

Kwanele Shishane, Johannes John-Langba & Eyitayo Onifade

Has been revised thoroughly for "English Editing," and the undersigned approves it for publishing

Signed: 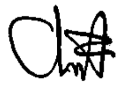

Jimmy J.

Email: oliviajayden009@gmail.com
